# Adaptive Immunity to *Francisella tularensis* and Considerations for Vaccine Development

**DOI:** 10.3389/fcimb.2018.00115

**Published:** 2018-04-06

**Authors:** Lydia M. Roberts, Daniel A. Powell, Jeffrey A. Frelinger

**Affiliations:** ^1^Immunity to Pulmonary Pathogens Section, Laboratory of Bacteriology, Rocky Mountain Laboratories, National Institute of Allergy and Infectious Diseases (NIAID), National Institutes of Health (NIH), Hamilton, MT, United States; ^2^Department of Immunobiology and Valley Fever Center for Excellence, University of Arizona, Tucson, AZ, United States

**Keywords:** *Francisella tularensis*, vaccine development, immune response, T cells, Antibodies

## Abstract

*Francisella tularensis* is an intracellular bacterium that causes the disease tularemia. There are several subspecies of *F. tularensis* whose ability to cause disease varies in humans. The most virulent subspecies, *tularensis*, is a Tier One Select Agent and a potential bioweapon. Although considerable effort has made to generate efficacious tularemia vaccines, to date none have been licensed for use in the United States. Despite the lack of a tularemia vaccine, we have learned a great deal about the adaptive immune response the underlies protective immunity. Herein, we detail the animal models commonly used to study tularemia and their recapitulation of human disease, the field's current understanding of vaccine-mediated protection, and discuss the challenges associated with new vaccine development.

## Introduction

*Francisella tularensis* is a Gram-negative intracellular bacterium and the causative agent of tularemia. *Francisella* can be transmitted by aerosol, breaks in the skin, ingestion of contaminated water, and bites of infected arthropods. Virulent, or Type A strains, of *F. tularensis* subspecies *tularensis* (*F. tularensis*) cause severe disease in both humans and other vertebrates, even infecting soil amoeba, at low exposure doses. The less virulent Type B *F. tularensis* subspecies *holartica* (*F. holartica*) strains also have a broad host range, but do not cause severe disease. While only 100–200 natural cases of tularemia are reported each year in the US, *F. tularensis* is a significant biothreat and has been weaponized (Christopher et al., 1997; Alibek and Handelman, 1999). Today, *Francisella* is categorized as a Tier 1 Select Agent due to its low infectious dose, ease of aerosolization, and ability to persist in the environment.

Ideally, there would be an efficacious vaccine for such a high consequence pathogen, however, no licensed vaccine for tularemia is available. The Live Vaccine Strain (LVS) was developed in the Soviet Union from *F. holartica* and provides limited protection (Eigelsbach and Downs, 1961). This vaccine is not currently licensed in the United States as the protection engendered is limited. Many recent attempts have been made to produce new vaccines against *Francisella*. While a successful vaccine has yet to be produced, the collective knowledge gained from these studies has provided many important insights into the immune response to *Francisella* vaccination and subsequent protection. Together, these data provide critical information as to the nature of protective immunity that must be provoked by future vaccine candidates.

Here, we discuss the animal models used to study the immune response to *Francisella* including their recapitulation of human disease and respective limitations. Next, we detail the adaptive immune response and the effector functions that have been identified as protective. Finally, we address the challenges associated with developing effective tularemia vaccines.

## Characteristics of human infection

Tularemia presents in human patients in several forms dependent on exposure route and subspecies of the infecting strain. The most common presentation is ulceroglandular tularemia which is generally caused by an arthropod bite or skin abrasions (Tärnvik et al., 1996; Ohara et al., 1998). Bacteria will spread from this entry site through the lymphatic system to draining lymph nodes. From the lymph nodes, bacteria may disseminate to the periphery including the spleen, liver, lungs, kidneys, central nervous system, and skeletal muscle (Ellis et al., 2002). Ulceroglandular tularemia associated with subspecies *holarctica* is rarely fatal, with less than a 3% case mortality (Evans et al., 1985). Comparatively, pneumonic tularemia is caused by subspecies *tularensis* and carries a mortality rate ranging from 30 to 60% in the absence of therapeutic intervention (Gill and Cunha, 1997). Patients generally present with flu-like symptoms including chills, fever and headaches; diagnosis is achieved by selective culture, PCR, or serology (Burke, 1977; Carlsson et al., 1979; Koskela and Salminen, 1985; Syrjälä et al., 1986; Clarridge et al., 1996; Johansson et al., 2000). Treatment with antibiotics, like ciprofloxacin, is generally effective although β-lactam antibiotics are not due to a β-lactamase gene in *Francisella*. Convalescent patients have detectable antibody and T cell responses which are described in more detail later.

## Animal models of tularemia

The 2002 “Animal Rule” (21 CFR 314.600-650 and 601.90) from the United States Food and Drug Administration (FDA) applies to development of novel *F. tularensis* therapeutics and vaccines given the highly pathogenic nature of human infection. The inability to ethically or appropriately test new therapies in humans requires efficacy testing in relevant animal model(s) prior to FDA licensure. Recently, a novel *Bacillus anthracis* vaccine was approved under the animal rule and several therapeutics for high consequence pathogens have been approved in the last decade after clinical efficacy was determined in appropriate animal models (Beasley et al., 2016; Park and Mitchel, 2016). There are multiple animal models for tularemia and their ability to recapitulate human disease is discussed below.

### Mice

The mouse is the most commonly used animal to study tularemia due to its relatively low cost, well-characterized genetics, and available immunological tools. Most importantly, mouse infection with virulent *F. tularensis* recapitulates human disease. Like humans, mice are extremely susceptible to low doses (< 50 CFUs) of *F. tularensis*, developing disseminated disease that is asymptomatic for the first 2–3 days after inoculation (Shen et al., 2004; Pechous et al., 2008). Additionally, mice and humans can be successfully vaccinated with *F. holaritca* LVS, but this protection only applies to low *F. tularensis* inoculum doses within a short timeframe post-vaccination (McCrumb, 1961; Saslaw et al., 1961a; Chen et al., 2003; Conlan et al., 2005; Roberts et al., 2017). Mice are more resistant to *F. holartica* than *F. tularensis* by certain routes of inoculation, yet extremely susceptible to *F. novicida* (Fortier et al., 1991; Conlan et al., 2003; Lauriano et al., 2004; Wu et al., 2005). Although the susceptibility of humans and mice differs greatly for *F. novicida* and there are some differences for *F. holartica*, these discrepancies are not critical as they relate to the animal rule. The animal rule applies only to *F. tularensis*; therefore, the animal model used to test novel vaccines or therapeutics only needs to closely resemble human disease with *F. tularensis*. The BALB/c and C57Bl/6 mouse strains are the most prevalent in the literature for evaluating immune responses to *Francisella* although a variety of common laboratory mouse strains were tested in Shen et al. (2004). Initially, only BALB/c mice could survive *F. tularensis* challenge after immunization with LVS (Shen et al., 2004; Wu et al., 2005; KuoLee et al., 2007; Twine et al., 2012). More recently, C57Bl/6 mice were protected using a different strain of LVS (RML LVS) indicating the vaccinating strain utilized is critical for the development of protective immunity (Griffin et al., 2015).

### Rats

Historically, white rats were used in tularemia studies and found to be more resistant to *F. tularensis* than mice when various inoculation routes were tested (Downs et al., 1947). More recently, Fisher 344 rats have been used and found to mimic human susceptibility to the various subspecies of *Francisella* (Ray et al., 2010). The *F. tularensis* intratracheal LD_50_ for Fisher 344 rats is ~500 CFUs which is higher than the 10–15 CFUs that can cause lethal disease in humans (McCrumb, 1961; Ray et al., 2010). Despite this moderate difference in susceptibility, pulmonary infection of rats does recapitulate human disease pathology (Francis and Callender, 1927; Dennis et al., 2001; Lamps et al., 2004; Guarner and Zaki, 2006; Hutt et al., 2017). *F. holartica* LVS and *F. novicida* vaccine efficacy has been evaluated in Fisher 344 rats and found to protect against virulent challenge (Wu et al., 2009; Signarovitz et al., 2012; Chu et al., 2014). One argument for the use of rats as the preferred small animal model is their ability to protected from high doses of pulmonary *F. tularensis* challenge (2 × 10^5^ CFU) after *F. holartica* LVS vaccination (Wu et al., 2009). While the ability to protect against high doses of *F. tularensis* is a primary goal in vaccine development, the rat's natural resistance to *F. tularensis* may overestimate the protective efficacy of a vaccine candidate as human studies have demonstrated poor or moderate protection with 10- to 100-fold lower challenge doses (McCrumb, 1961; Hornick and Eigelsbach, 1966).

### Rabbits

The use of rabbits as an animal model for tularemia has recently been revisited. Tularemia is also known as “rabbit fever” and rabbits are a natural host for *Francisella* species. Disease in the rabbit recapitulates human pathology and rabbits show similar susceptibility to the different subspecies of *Francisella* like humans (Baskerville and Hambleton, 1976; Reed et al., 2011; Brown et al., 2015a). New Zealand White rabbits tolerate high doses of *F. holartica* LVS during oral, respiratory, or scarification vaccination, yet vaccinated animals do not survive *F. tularensis* challenge (Pasetti et al., 2008; Reed et al., 2014; Stinson et al., 2016). Similarly, type B vaccinated wild-caught cottontail rabbits had an extension in the mean time to death after type A challenge compared to unvaccinated animals but did not survive virulent secondary infection (Brown et al., 2015b). Defined *F. tularensis* mutants were partially protective against aerosol challenge with 50–500 LD_50_ doses of wild-type *F. tularensis* in the New Zealand White rabbit suggesting the choice of vaccinating strain impacts protection (Reed et al., 2014). Overall, the rabbit is another appropriate small animal model for evaluating vaccine efficacy prior to non-human primate (NHP) or human studies.

### Non-human primates

Although NHP studies are more challenging and costly to conduct, this animal model also recapitulates tularemia pathology in humans. Importantly, NHPs mirror several aspects of human disease not observed in the rabbit, rat, or mouse. First, NHPs can develop skin lesions and lymphadenopathy (Nelson et al., 2010). Second, primates have V9γV2δ T cells which expand after human infection, but are absent in small rodents (Sumida et al., 1992; Kroca et al., 2000). Several NHP species have been used in tularemia studies including African green monkeys, cynomolgus macaques, grivet monkeys, rhesus macaques, and marmosets (Hornick and Eigelsbach, 1966; Sawyer et al., 1966; Tulis et al., 1970; Baskerville et al., 1978; Hambleton et al., 1978; Nelson et al., 2009, 2010; Twenhafel et al., 2009; Chu et al., 2014; Glynn et al., 2015). Most NHP species have similar susceptibility to *F. tularensis* infection as humans with lethal infectious doses <100 CFUs (Nelson et al., 2009; Glynn et al., 2015). While the LD_50_ for rhesus macaques was determined to be low (14 CFU) in the 1970's, a more recent study found they were remarkably resistant (lethal dose >2 × 10^5^ CFU) (Day and Berendt, 1972; Glynn et al., 2015). The original study found the particle size affected the LD_50_ with larger particles having higher LD_50_ values (Day and Berendt, 1972). This factor could be contributing to the large difference in LD_50_ values between the two studies. There have been a limited number of vaccine studies in NHP using either LVS or *F. novicida*. As observed in the mouse and rat, LVS vaccination can protect NHP during *F. tularensis* challenge (Eigelsbach et al., 1962; White et al., 1962; Hornick and Eigelsbach, 1966; Chu et al., 2014). To date, there is no consensus on the most appropriate NHP species to use for tularemia studies although there are clearly several candidates that mirror human disease.

Ultimately, studies in mice, rats, rabbits, and NHPs will likely be required to satisfy the Animal Rule for new tularemia vaccines or therapeutics. Mice, rats, and rabbits are particularly useful for evaluating vaccine efficacy and defining mechanisms of protection given their small size, available tools, and ability to recapitulate various aspects of human disease. A vaccine or therapeutic that is successful in small mammals, especially given the mouse's increased susceptibility to *F. tularensis*, is likely to have success in NHPs. Following confirmatory studies in NHPs that indicate a high probability of success in humans, the FDA's Animal Rule will be satisfied.

## Immune responses to *Francisella*

### B cells

Tularemia infection induces anti-*Francisella* antibodies in both mouse and man (Koskela and Herva, 1982; Koskela, 1985; Koskela and Salminen, 1985; Janovská et al., 2007). Many of these antibodies are directed against the LPS components, especially early in the infection, but many other immunogenic proteins have been described (Dreisbach et al., 2000; Eyles et al., 2007). It was reported that immunization of DBA/2 and C57Bl/6 with *F. holartica* LVS did not protect mice from lethal challenge with virulent *F. tularensis*. In contrast, vaccination of BALB/c or C3H/HeN mice were protected following identical vaccination (Twine et al., 2006b; Kilmury and Twine, 2010). Serum from C57Bl/6 and BALB/c were shown to recognize both shared and unique proteins from *Francisella*. It is not clear if this reflects an intrinsic difference in their B cell responses or a difference in the CD4 helper response. The proteins differentially recognized include outer membrane associated proteins as well as protein chain elongation factors (Twine et al., 2006a).

Antibodies against *Francisella* LPS have shown a protective capacity against lethal intradermal and intraperitoneal LVS challenge (Rhinehart-Jones et al., 1994; Culkin et al., 1997; Fulop et al., 2001; Stenmark et al., 2003; Sebastian et al., 2007). This protection is induced early after challenge and is driven by poly-specific IgM against the LPS components, though non-specific stimulation with monophosphoryl lipid A (MPL) could provide similar protection against LVS challenge (Cole et al., 2011). Given that intradermal vaccination with *F. holartica* LVS does not provide protection against *F. tularensis* intranasal challenge, and that intranasal vaccination protects against both routes of challenge suggests mucosal IgA could be involved (Conlan et al., 2005; Wu et al., 2005). IgA has been detected in the serum of both humans and mice as well as BAL from vaccinated mice (Koskela and Herva, 1982; Koskela, 1985; Koskela and Salminen, 1985; Lavine et al., 2007; Rawool et al., 2008). The protective effect of anti-*Francisella* antibodies (subclass undefined) has been shown to be independent of complement yet dependent on Fc receptors and phagocytosis (Kirimanjeswara et al., 2007).

Early treatments for *Francisella* centered around the use of immune serum (Francis and Felton, 1942; Foshay, 1950; Tärnvik, 1989). It is unclear whether this treatment was effective against pulmonary tularemia (Kirimanjeswara et al., 2008). In mice, serum transfer shows some protection against pulmonary *F. holartica* LVS and *F. novicida* infection (Pammit et al., 2006; Lu et al., 2007). Serum transfer from *F. holartica* LVS-immune animals provides no protection against *F. tularensis* pulmonary infection in BALB/c mice (Kirimanjeswara et al., 2008). In another model, convalescent serum from an *F. tularensis*-infected levofloxacin treated mouse was protective in BALB/c mice (Klimpel et al., 2008). The protection provided by antibody transfer was dependent on FcγR-mediated opsonophagocytosis as well as ADCC by Natural Killer cells (Kirimanjeswara et al., 2007; Sanapala et al., 2012). Additionally, it is important to note that the protective ability of transferred serum is dependent on T cells in both the mouse and rat (Kirimanjeswara et al., 2008; Mara-Koosham et al., 2011). Therefore, the protection seen in these models is likely a consequence of an intact T cell response. Recently, nanoparticles incorporating lysates from either LVS or SchuS4, along with MPL have be shown to protect mice from lethal LVS challenge (Richard et al., 2017). This regime resulted in both an augmented T cell INF-γ response as well as an increased antibody response. The impact of these responses separately has not been determined.

While the ability to detect anti-*Francisella* antibodies is an indicator of previous exposure, antibody titers are poor predictors of a vaccine's protective efficacy in humans (Saslaw et al., 1961a,b). As a pathogen that prefers to replicate intracellularly, *Francisella* is typically inaccessible to the antibody response. However, the organism can be found extracellularly and thus antibodies could play a role in controlling infection (Forestal et al., 2007; Yu et al., 2008). The demonstration by several groups that T cells are required for immune sera to be protective suggests that antibodies buy the host time for the T cell response to appropriately develop. Further, B cells have been shown to play an important antibody-independent role during secondary *F. holartica* LVS infection as antigen-presenting cells and/or cytokine producers (Elkins et al., 1999). Therefore, while measuring the antibody response is a straightforward measure of *Francisella* exposure, vaccine development should focus on understanding the protective T cell response.

### T cells

#### αβ T cells

Decades of *Francisella* research have demonstrated the absolute requirement for T cells for the clearance of primary infections and protective immunity. Mice lacking T cells such as αβ TCR^−/−^ or *nu*/*nu* mice develop a chronic *F. holartica* LVS infection that is eventually lethal (Elkins et al., 1993, 1996; Yee et al., 1996). Although naïve mice succumb to *F. tularensis* infection prior to the development of adaptive immunity, a convalescent model of *F. tularensis* infection shows αβ TCR^−/−^ and SCID mice succumb to infection after antibiotic treatment is halted (Crane et al., 2012). T cells are also key mediators of protective immunity in both homotypic and heterotypic vaccination and challenge models (Yee et al., 1996; Chen et al., 2004; Conlan et al., 2005; Wu et al., 2005; Mara-Koosham et al., 2011; Roberts et al., 2016). Depletion of either CD4^+^ or CD8^+^ T cells in immune animals prior to *F. tularensis* challenge eliminates protective immunity in both BALB/c and C57Bl/6 mice with slight differences in mean time to death kinetics. Immune BALB/c mice lacking either CD4^+^ or CD8^+^ T cells have similar mean time to death whereas C57Bl/6 mice depleted of CD4^+^ T cells succumb to *F. tularensis* significantly faster than animals depleted of CD8^+^ T cells (Conlan et al., 2005; Roberts et al., 2016). These data indicate that both subsets of T cells are required for protective immunity with slightly different requirements depending on the mouse and vaccinating strain. The critical role of CD4^+^ T cells in C57Bl/6 mice is likely a consequence of the immune response being dominated by CD4^+^ T cells with at least 2-fold more cells during or after vaccination compared to CD8^+^ (Cowley et al., 2005; Woolard et al., 2008; Barrigan et al., 2013; Griffin et al., 2015).

#### γδ T cells

While αβ T cells are critical during primary and secondary infection with *Francisella*, γδ T cells are dispensable. γδ TCR^−/−^ mice are not more susceptible to primary intranasal or intradermal infection with *F. holartica* LVS (Yee et al., 1996; Markel et al., 2010). In a convalescent model of *F. tularensis*, γδ TCR^−/−^ mice are not more susceptible than wild-type mice during the primary or secondary challenge (Crane et al., 2012). Together, γδ T cells do not play a major role in resolving *Francisella* infection in the mouse. However, Vγ9/Vδ2 T cells comprise almost all peripheral γδ T cells in infected humans and can make up one-third of all CD3^+^ T cells 1 month after infection (Sumida et al., 1992; Poquet et al., 1998). Purified γδ T cells from some human patients are capable of controlling *F. holartica-*LVS replication in THP-1 cells by an IFN-γ-dependent mechanism (Rowland et al., 2012). There is evidence that γδ T cells produce cytokines after infection (discussed below) and therefore are contributing to the immune response albeit at a lower level than other T cell subsets.

#### CD4^−^ CD8^−^ double negative T cells

Mucosal associated invariant T cells (MAITs) are characterized by the lack of CD4 and CD8 expression and are MHC-related protein 1-restricted. Mice depleted of CD4^+^ and CD8^+^ T cells during *F. holartica* LVS infection are chronically infected suggesting this MAIT population controls bacterial burdens, but does not mediate clearance (Yee et al., 1996; Meierovics et al., 2013). MAITs are preferentially located in the lungs of intranasally inoculated mice, contribute to monocyte differentiation into dendritic cells, and support the response of CD4^+^ and CD8^+^ conventional T cells (Meierovics et al., 2013; Meierovics and Cowley, 2016). While it is clear MAITs play a role during attenuated *F. holartica* LVS infection, their contribution to virulent *Francisella* infection has not been evaluated.

### Important T cell effector functions

Identifying the effector function(s) necessary for controlling infection is a critical aspect of vaccine development. T cells from convalescent humans produce IFN-γ, TNF-α, IL-2, IL-17, and IL-22 indicating these cytokines are elicited by natural infection or vaccination and therefore should be further assessed for their role in protective immunity in animal models (Karttunen et al., 1991; Surcel et al., 1991; Ericsson et al., 1994; Salerno-Gonçalves et al., 2009; Paranavitana et al., 2010; Eneslätt et al., 2012). The requirement of the classical Th1 cytokines IFN-γ and TNF-α during murine *Francisella* infection has been demonstrated by multiple groups (Leiby et al., 1992; Sjöstedt et al., 1996; Collazo et al., 2006, 2009; Crane et al., 2012; Skyberg et al., 2013; Roberts et al., 2014). *F. holartica* LVS is highly susceptible to IFN-γ (Anthony et al., 1989; Fortier et al., 1992). *In vitro*, IFN-γ directly controls *F. holartica* LVS replication in peritoneal macrophages using a reactive-nitrogen dependent mechanism (Fortier et al., 1992). However, in alveolar macrophages, IFN-γ control of *F. holartica* LVS is reactive nitrogen and TNF-α independent (Polsinelli et al., 1994). Further, pre-treatment of mouse or human macrophages with IFN-γ controls *F. tularensis* infection via reactive nitrogen and reactive oxygen independent mechanisms (Edwards et al., 2010). Together these data suggest the role of reactive nitrogen and oxygen species is cell-type dependent and another unknown mechanism to restrict intracellular growth exists. In another model of *in vitro F. tularensis* infection of mouse macrophages, treatment with IFN-γ alone after infection did not control bacterial replication (Roberts et al., 2016). Instead, both IFN-γ and TNF-α were required (Roberts et al., 2016); the mechanism(s) that underlie IFN-γ and TNF-α control of *F. tularensis* in BMMs has not yet been elucidated. However, the requirement for both effector cytokines for controlling bacterial replication indicate that a vaccine candidate should elicit poly-functional T cells to maximally control *F. tularensis* infection. IL-17A is also produced by CD4^+^, CD4^−^ CD8^−^ double negative, and γδ T cells following pulmonary infection with *F. holartica* LVS, but absent when animals are peripherally inoculated (Woolard et al., 2008; Cowley et al., 2010; Markel et al., 2010). Mice deficient in IL-17 are more susceptible to primary infection with *F. holartica* LVS, yet IL-17 is dispensable during secondary infection with either *F. holartica* LVS or *F. tularensis* (Woolard et al., 2008; Lin et al., 2009; Cowley et al., 2010; Markel et al., 2010; Skyberg et al., 2013; Roberts et al., 2014).

The ability of CD4^+^ and CD8^+^ T cells to produce cytokines after vaccination or challenge has been evaluated using ELISPOT, ELISA, and intracellular cytokine staining. These tried and true methods are appropriate in many situations but are not a direct measure of a specific cell population's ability to control intracellular replication. One technique used by multiple laboratories to directly assess immune cell function is to co-culture infected bone marrow macrophages (BMMs) with a population of interest, e.g., CD4^+^ T cells. This technique has been used to determine whether specific cell populations are capable of mediating bacterial control and if so, what molecular mechanisms are required (Cowley and Elkins, 2003; Cowley et al., 2005; Collazo et al., 2009). Using this technique, groups have demonstrated that CD4^+^, CD8^+^, and MAIT cells control attenuated or virulent *Francisella* replication in macrophages, further underscoring the importance of these cell subsets during infection (Cowley and Elkins, 2003; Cowley et al., 2005; Collazo et al., 2009; Roberts et al., 2016). Although the control of bacterial replication is mostly dependent on IFN-γ, several groups have demonstrated a small, but significant degree of IFN-γ independent control of *F. holartica* LVS replication in macrophages (Cowley and Elkins, 2003; Collazo et al., 2009). IFN-γ-independent control of *Francisella* infection could be a result of cytotoxic activity. Unfortunately, the contribution of granzyme B and/or perforin has not been evaluated in *F. holartica* LVS or *F. tularensis* infection. Perforin does contribute to protection after *F. novicida* vaccination and was necessary for primed T cells to optimally control bacterial replication in macrophages (Sanapala et al., 2012). Co-culture assays have also been used to identify correlates of protection and vaccine efficacy (De Pascalis et al., 2012, 2014; Griffin et al., 2015; Golovliov et al., 2016; Roberts et al., 2016, 2017). Thus far, the identified correlates of protection are consistent with our understanding of protective immunity and include classic Th1-associated responses (IFN-γ, IL-12, and T-bet) as well as IL-6, IL-18, SOCS-1, and iNOS (De Pascalis et al., 2012; Golovliov et al., 2016). Overall, the use of a co-culture system to define the mechanism of protection will likely be an important component of vaccine evaluation and is a useful *in vitro* system to screen vaccine candidates. Furthermore, co-culture assays can be used to determine the ability of human immune cells to control *F. tularensis* replication and confirm mechanisms of protection discovered in animal models.

### Route of vaccination and influence on the immune response

The route of vaccination, bacterial strain, and mouse strain utilized has a strong influence on whether a vaccine candidate is deemed protective. For example, while mice vaccinated via the intradermal route with *F. holartica* LVS are protected only against subsequent intradermal challenge, no protection against pulmonary *F. tularensis* challenge is provided (Wu et al., 2005; KuoLee et al., 2007). Using another strain of *F. holartica* LVS, Anderson, et al. demonstrated BALB/c mice are protected from pulmonary *F. tularensis* challenge after subcutaneous vaccination (Anderson et al., 2010). Further, mice vaccinated intranasally are protected against challenge by either the intradermal or intranasal route, suggesting the location of the protective cell is important. When considering the development of protective T cell responses, it is therefore important to understand the localization of protective T cells. Tularemia is a disseminated disease, causing T cells to respond throughout the body during primary and secondary infection. Not surprisingly, the location of T cells during and after vaccination differs depending on the mouse strain and route of vaccination. A direct comparison was made between C57Bl/6 mice intradermally and intranasally vaccinated with *F. holartica* LVS. The CD4^+^ T cell response in the spleen and lung more rapidly expands after intradermal vaccination whereas T cells are only present in the broncheoalveolar lavage fluid after intranasal vaccination (Woolard et al., 2008). In a prime-boost model of intranasal *F. holartica* LVS vaccination in C57Bl/6 animals, the number of effector and cytokine-producing CD4^+^ T cells in the lung is significantly increased compared to prime only, whereas there is no difference in the spleen (Roberts et al., 2017). These data suggest multiple intranasal exposures specifically boost the number of T cells in the pulmonary compartment. In contrast, protection in immune BALB/c mice challenged intranasally with *F. tularensis* correlated with splenic activated and cytokine-producing CD4^+^ T cells as opposed to pulmonary T cells (Anderson et al., 2010). The difference in protective T cell location between BALB/c and C57Bl/6 is likely a mouse strain difference but highlights the importance of understanding the location of protective T cells in tularemia. Specifically, C57Bl/6 mice are not protected 90 days after a single LVS vaccination whereas BALB/c mice are (Anderson et al., 2010; Roberts et al., 2017).

### T cell epitopes

*F. holartica* LVS is not licensed for use in the United States and it is unlikely that any live vaccine will be licensed for tularemia due to safety concerns. Generation of an acellular vaccine will require the identification of epitopes recognized by the adaptive immune system combined with adjuvants that provoke the appropriate T cell response. The ability of a vaccine to provoke high avidity CD4^+^ T cells significantly improves vaccine efficacy (Roberts et al., 2016, 2017). While this system uses an epitope not present in *Francisella*, it serves as proof-of-concept that identifying this class of epitope is critical for future acellular vaccine development. Several CD4^+^ epitopes have been identified in the mouse, including the C57Bl/6 immunodominant epitope, LpnA_86−99_, which comprises up to 20% of responding CD4^+^ T cells after LVS infection (Valentino et al., 2009, 2011). A computational approach was taken to identify CD8-resistricted epitopes and a DNA-based vaccination containing the most prominent epitopes did protect during *F. holartica* LVS challenge (Rotem et al., 2014). Bioinformatics also identified *Francisella* peptides with predicted binding to human MHCI and MHCII (McMurry et al., 2007). The response to these peptides was then tested in PBMCs from humans previously infected with *F. tularensis* using ELISPOT and 39 novel epitopes were identified (McMurry et al., 2007). A comprehensive list of proteins recognized by convalescent human sera and *F. holartica* LVS-vaccinated mouse serum is presented in Kilmury and Twine (2010). This list is particularly useful because recognition of a protein by immune sera strongly suggests a T cell epitope is also present in that protein. In addition to being recognized by human sera, LpnA is recognized by sera from vaccinated NHP, rats, and mice suggesting T cell epitopes recognized by multiple species are present in this protein (Havlasová et al., 2002; Eyles et al., 2007; Chu et al., 2014). Given the diversity of MHC alleles across species and the requirements of the Animal Rule, *Francisella* proteins that evoke immune responses in mice, rats, NHPs, and humans like LpnA are attractive vaccine targets.

## Rational vaccine design

Many labs have investigated potential vaccines by screening the ability of mutant *Francisella* strains that do not cause disease themselves to act as a live vaccine (reviewed in Conlan, 2011; Marohn and Barry, 2013). In many cases, these strains offer the same or enhanced protection compared to wild-type *F. holartica* LVS. Instead of targeting strains that are attenuated for growth as vaccine candidates, our lab has used a different approach. We found that *Francisella* infected macrophages produce prostaglandin E2 (PGE_2_) that blunts the T cell IFN-γ response (Woolard et al., 2007, 2008). Mice treated with indomethacin to inhibit PGE_2_ production had lower bacterial loads indicating the bacterium is manipulating the host immune response to its benefit. Therefore, instead of using a screen to find growth-attenuated bacteria, we identified an immune evasion trait of *Francisella* and selected mutants that were unable to suppress that particular immune response. When we screened a *F. novicida* mutant library, we found that mutants in the *clpB* gene were unable to induce PGE_2_ secretion in infected macrophages (Woolard et al., 2013). Upon further study, we found that *F. holartica* LVS carrying mutations in this gene were attenuated *in vivo*, rarely produced disease, and protected against a lethal wild-type *F. holartica* LVS challenge (Barrigan et al., 2013). Similarly, an *F. tularensis* ΔclpB mutant is also attenuated *in vivo* yet elicits a protective immune response during wild-type *F. tularensis* challenge (Conlan et al., 2010; Twine et al., 2012). The experiments described above clearly show that we can identify mutations that attenuate *Francisella* infection without directly affecting bacterial growth *in vitro*. Therefore, it is important to also consider mutations that target immune evasion mechanism(s) as potential vaccine candidates.

Although a live vaccine for tularemia may induce a protective immune response, safety concerns may ultimately prevent licensure. In lieu of live attenuated strains as vaccine candidates, several groups have investigated the use of acellular tularemia vaccines including glycoconjugate vaccines, purified outer membrane proteins, immune stimulating complexes, and catanionic surfactant vesicles (Golovliov et al., 1995; Huntley et al., 2008; Cuccui et al., 2013; Richard et al., 2014, 2017). These acellular vaccines evoke partial protection when animals were challenged with *F. tularensis*. Identification of protective antigens will significantly improve the development of new acellular *Francisella* vaccines and should be a focus of future research.

Irrespective of the vaccine choice, an important consideration for its development will be the vaccination route(s). As discussed above, the route of vaccination influences the location and function of immune T cells (Woolard et al., 2008). The ability of *Francisella* species to cause disease via a variety of routes and the disseminated nature of tularemia suggests that the most effective vaccination strategy will provoke T cells in a variety of tissues. One mechanism to provoke multiple pools of protective T cells is to utilize a prime/boost strategy where one immunization is done via inhalation and one intradermally or subcutaneously. This approach will quantitatively improve the immune response while inducing memory T cells in multiple tissues.

Novel vaccine candidates are likely to be tested first in mice prior to moving to other small mammals and eventually NHPs. The mouse is the most rational choice for initial studies because of the immunological tools available to clearly define mechanisms of protection. To date, the identified mechanisms of protection are the same between mice, rats, and man therefore there is a high likelihood that results from a novel vaccine candidate will translate to humans. A final critical consideration for vaccine development is the requirement that a candidate be evaluated for its ability to protect against pulmonary infection with *F. tularensis*. While we have learned a great deal about the immune response during tularemia using homologous vaccine and challenge studies, challenge with *F. tularensis* is the most rigorous evaluation of a vaccine candidate's ability to elicit a protective immune response.

## Future challenges

Considerable progress has been made in understanding aspects of protective immunity to *Francisella*, yet important challenges remain. First, vaccines tested to-date only protect against low to moderate pulmonary challenges with *F. tularensis* in both mice and man (McCrumb, 1961; Saslaw et al., 1961a; Chen et al., 2003; Conlan et al., 2005; Roberts et al., 2017). The difficultly protecting against higher respiratory doses may be a consequence of an insufficient T cell response and/or the unique ability of *F. tularensis* to inhibit the innate immune response (Bosio et al., 2007; Crane et al., 2013a,b; Gillette et al., 2014). Higher inoculum doses result in more bacteria interacting with target cells and potentially a more complete inhibition of innate immunity. Without the proper innate immune signals, T cells are not activated until bacterial loads are too high to overcome. Even with low inoculum doses, protective immunity to *F. tularensis* wanes quickly (Burke, 1977). Therefore, another challenge of vaccine development will be to provoke long-lasting central memory cells. The inability to protect mice against high challenge doses for long periods of time makes them an ideal model for testing vaccine candidates. Further, the genetic and immunological tools available for the mouse allow the protective immune response to be defined. Ultimately, success in multiple animal models will be required for approval of novel tularemia vaccines or therapeutics under the Animal Rule.

During the last 10 years there has been remarkable improvement in our understanding the immune response to *Francisella*. This has been accompanied by production of a wide variety of potential vaccines, ranging from those developed using classical vaccinology, attenuated live bacteria, immunization using novel nanoparticles, and even LPS. Our own work has focused on better understanding protective immunity to *Francisella*, from defining mechanisms of immune evasion that can be modulated to more recent work identifying correlates of protection during *F. tularensis* challenge in immune animals (Woolard et al., 2007, 2008; Roberts et al., 2016, 2017). Even if live attenuated bacteria are never licensed for use, our understanding of immunity *Francisella*, and potentially other pulmonary bacterial pathogens have been greatly expanded.

## Author contributions

All authors listed made substantial direct and intellectual contributions to the work and approved it for publication.

### Conflict of interest statement

The authors declare that the research was conducted in the absence of any commercial or financial relationships that could be construed as a potential conflict of interest.
